# Use of the TIDieR checklist to describe an online structured education programme for type 2 diabetes

**DOI:** 10.1177/2055207620975647

**Published:** 2020-11-30

**Authors:** Shoba Poduval, Jamie Ross, Kingshuk Pal, Nicola Newhouse, Fiona Hamilton, Elizabeth Murray

**Affiliations:** 1 Department of Primary Care & Population Health, 4919 University College London, Royal Free Campus, London, UK; 2 Nuffield Department of Primary Care Health Sciences, University of Oxford, Radcliffe Observatory Quarter, Oxford, UK

**Keywords:** Type 2 diabetes, patient self-management, diabetes education, primary care, digital health

## Abstract

**Objectives:**

The aim of structured education for type 2 diabetes is to improve knowledge, skills and confidence in self-management. It is recommended in the UK for everyone who is newly diagnosed with type 2 diabetes. We developed an on-line programme called HeLP-Diabetes: Starting Out to address poor uptake of face-to-face structured education. The aim of this paper is to describe the intervention in line with the Template for Intervention Description and Replication guide, which calls for better reporting of interventions.

**Methods:**

The Template for Intervention Description and Replication guide provided the item headings for the description. These included the theoretical underpinning, materials, procedures, providers, and mode of delivery.

**Results:**

The programme was developed to meet NICE requirements for structured education and therefore followed a structured curriculum with four sessions covering content such as what diabetes is and how it is treated, possible complications, and how lifestyle changes can improve health. Content was delivered in text, images and video, and behaviour change techniques, self-assessment and feedback were used to help people target key health behaviours. The programme was delivered entirely online, but the team were available for support via telephone. Email feedback and reminders were sent.

**Conclusions:**

The TIDieR checklist allowed us to provide a clear structure for the description of the intervention. However, it could not capture the full complexity of the programme, and intervention developers considering using it in the future may find that it needs to be adapted to make it more specific to their intervention.

## Introduction

### Rationale for the programme

The aim of structured education for Type 2 Diabetes Mellitus (T2DM) is to improve people’s knowledge, skills and confidence to take control of their condition and integrate self-management into daily life.^1^ Self-care behaviours in T2DM have been defined by the American Association of Diabetes Educators as healthy eating, physical activity, blood glucose monitoring, medication adherence, problem-solving skills, coping skills and risk-reduction behaviours.^2^ In the United Kingdom (UK) standards for diabetes education have been set by the Diabetes UK (DUK) Patient Education Working Group in collaboration with the Department of Health.^1^ Structured patient education is recommended by the National Institute for Health and Care Excellence (NICE) for everyone with T2DM (and/or their carers) at and around the time of diagnosis.^3^ DUK and NICE recommend that education programmes should have a structured written curriculum, be evidence-based, theory-driven, and have specific aims and objectives.^3^ However, despite these policy initiatives, and financial incentives (through the Quality and Outcomes Framework or QoF) for primary care teams to refer patients to structured education, uptake remains low. National Audit Office data suggest than only 8.3% of eligible patients attended in 2016.^4^

Hence, improving uptake of structured education is a priority for the National Health Service (NHS) in England. Recommendations for improving uptake have been made, and this includes offering patients online courses.^5–7^ Systematic reviews of online self-management support have been conducted suggesting evidence of improvements in glycaemic control and self-care behaviours.^8–11^

A National Institute for Health Research (NIHR) programme grant funded the development of an online self-management resource called HeLP-Diabetes for people with T2DM. Randomised controlled trial (RCT) data showed this to be effective and cost-effective,^12^ but it was aimed at patients at all stages of the illness journey, not just newly diagnosed patients, and hence did not follow a structure with specific aims and objectives, and a structured written curriculum, as recommended by NICE^3^ and required for certification by the Quality Institute for Self-Management Education & Training (QISMET).^13^ Such certification was required for referral to the programme to be eligible for QOF remuneration. A structured programme for people newly diagnosed with T2DM (HeLP-Diabetes: Starting Out, or HDSO) was therefore developed in order to be NICE and QiSMET compliant. The key differences between HeLP-Diabetes and HeLP-Diabetes: Starting Out were:

- HeLP-Diabetes: Starting Out was aimed at people newly diagnosed with type 2 diabetes, rather than people at any stage of the illness.
- HeLP-Diabetes: Starting Out consisted of selected content from the HeLP-Diabetes website, and additional behaviour change techniques, relevant to people newly diagnosed with type 2 diabetes. It was therefore much smaller in size than HeLP-Diabetes.
- HeLP-Diabetes: Starting Out followed a structured spiral curriculum, with specific aims and learning objectives. People built on their learning as they worked through the programme. HeLP-Diabetes contained a large amount of information divided into sections, but without a curriculum or learning objectives.
- People worked through the modules in HeLP-Diabetes: Starting Out in a linear fashion, with access to the next module being given once the previous one had been completed. This is in contrast to HeLP-Diabetes, where people could dip in and out of different pages and sections depending on interest.

### Aim and objectives

Reviews of behaviour change interventions^14^ and online self-management interventions for TDM^15^ have found reporting of interventions to be limited. This results in less reliable implementation and replication of interventions. The aim of this paper is to describe the HeLP-Diabetes: Starting Out programme in line with the TIDieR (Template for Intervention Description and Replication) guide.^16^ A detailed description of the HeLP-Diabetes website is given elsewhere.^17^

## Methods

### Choice of checklist

The TIDieR checklist was developed in response to the poor quality of descriptions of interventions in publications. The TIDieR checklist is based on a literature review of relevant checklists and research, a Delphi survey of an international panel of experts, and the 2010 Consolidated Standards for Reporting Trials (CONSORT) and 2013 Standard Protocol Items: Recommendations for Interventional Trials (SPIRIT) statements. It consists of 12 items including: the name of the intervention; the rationale, theory or goal; the procedures, activities and processes used in the intervention; the intervention provider; the mode and location of delivery; the number of sessions the intervention is delivered in; if the intervention is personalised; if any modifications were made; if adherence was assessed, and the extent to which the intervention was delivered as planned (see Table 1).

**Table 1.** Items included in the Template for Intervention Description and Replication (TIDieR) checklist: Information to include when describing an intervention.

Item number	Item
Brief Name	
1	Provide the name or a phrase that describes the intervention
Why	
2	Describe any rationale, theory, or goal of the elements essential to the intervention
What	
3	Materials: Describe any physical or informational materials used in the intervention, including those provided to participants or used in intervention delivery or in training of intervention providers. Provide information on where the materials can be accessed (such as online appendix, URL)
4	Procedures: Describe each of the procedures, activities, and/or processes used in the intervention, including any enabling or support activities
Who provided	
5	For each category of intervention provider (such as psychologist, nursing assistant), describe their expertise, background, and any specific training given
How	
6	Describe the modes of delivery (such as face to face or by some other mechanism, such as internet or telephone) of the intervention and whether it was provided individually or in a group
Where	
7	Describe the type(s) of location(s) where the intervention occurred, including any necessary infrastructure or relevant features
When and how much	
8	Describe the number of times the intervention was delivered and over what period of time including the number of sessions, their schedule, and their duration, intensity, or dose
Tailoring	
9	If the intervention was planned to be personalised, titrated or adapted, then describe what, why, when, and how
Modifications	
10	If the intervention was modified during the course of the study, describe the changes (what, why, when, and how)
How well	
11	Planned: If intervention adherence or fidelity was assessed, describe how and by whom, and if any strategies were used to maintain or improve fidelity, describe them
12	Actual: If intervention adherence or fidelity was assessed, describe the extent to which the intervention was delivered as planned

Little guidance is available from journals about how to report interventions.^18^The TIDieR checklist was chosen for use in this paper because it provides a clear structure for describing interventions, with items covering the what, who, how, and when of an intervention and its delivery package, to permit successful implementation and replication. The items in the TIDieR checklist were particularly relevant to HeLP-Diabetes: Starting Out because it was delivered online in a number of sessions, by a multidisciplinary team. Using the checklist was therefore considered the most appropriate way to meet the aim of the paper.

### Choice of items and subcategories

This paper provides the information required by items 1–10. Items 11–12 (‘How well’) are discussed elsewhere.^19^ We have ensured that all items and relevant details are included by using items from the checklist as section headings in the results below. Subcategories reflect the description of the items given in the checklist as closely as possible. The section headings and subcategories used to describe the intervention are illustrated in Table 2, for ease of navigation for the reader.

**Table 2.** Item headings and subcategories used to describe the intervention.

Item heading	Subcategory
Brief name	
Why	Theoretical underpinning
What	Materials used to deliver intervention
	Information provided to participants (programme content)
	Procedures
Who provided	
How	Modes of delivery
Where	
When and how much	Spiral curriculum
	Session descriptions
Tailoring	Self-assessment
	Personalized feedback
	Personalized emails
	Goal-setting, action-planning and reviewing goals
Modifications	
How well	

**Table 3.** Session titles and parts.

Session titles	Session parts
Week 1 – Getting Started	Part 1 – An introduction to diabetes
	Part 2 – Self-assessment
	Part 3 – Eating well for diabetes
	Part 4 – Becoming more active
Week 2 – Self-management	Part 1 – Taking control
	Part 2- Protecting my body and mind
	Part 3 – Handling feelings
	Part 4 – Making changes
Week 3 – Improving my health and wellbeing	Part 1 – Making the most of the NHS
	Part 2 – Medication
	Part 3 – Reducing the risks of heart attack and strokes
	Part 4 – Update my goals and plans
	Part 5 – Understanding my moods
Week 4 – Taking control of my diabetes	Part 1 – My diabetes review
	Part 2 – Looking after my feet
	Part 3 – Review my goals and plans
	Part 4 – Self assessment
	Part 5 – Moving on: the end of the beginning
Week 5 – Bonus Content	Part 1 – Working with Health Professionals
	Part 2 – Diabetes and my social life
	Part 3 – Working with diabetes
	Part 4 – Driving with Diabetes
	Part 5 – Review my goals and plans
	Part 6 – Managing my moods

**Table 4.** Example feedback for the Diabetes Management Self-Efficacy Scale.

Lowest scoring area	Feedback
*Nutrition specific and weight*• *Managing my eating and weight.*	There is plenty of information to help you decide which foods to eat to manage your weight. We will cover some of this in session 1, and you can find more information in the “Staying Healthy” section of the HeLP-Diabetes website. You can also discuss diet with the Practice Nurse at your surgery.

## Results

### Brief name

The name of the intervention was HeLP-Diabetes (Healthy Living for People with Diabetes): Starting Out. HeLP-Diabetes was a website which people at any stage of their T2DM could use flexibly (this is described briefly in the background above and in more detail elsewhere).^17^ HeLP-Diabetes was followed by HeLP-Diabetes: Starting Out (HDSO), a structured education programme based on the content of the original website, but with a curriculum and learning objectives and aimed at newly diagnosed patients (Starting Out). This paper describes HeLP-Diabetes: Starting Out.

### Why

#### Theoretical underpinning

The theoretical model that was used to underpin HeLP-Diabetes was the Corbin and Strauss model of the work of managing a long term condition.^20^ This is conceptualised as consisting of three tasks: (1) medical management (adopting healthy behaviours, working with health professionals); (2) emotional mangement (addressing the negative emotions associated with being diagnosed with a long-term condition); and (3) role management (coming to terms with disruption to one's sense of self).

The aim of the HDSO programme was to help people newly diagnosed with type 2 diabetes to improve their knowledge, self-efficacy and emotional wellbeing by learning about how to address a wide range of needs including lifestyle changes, taking medication, interacting with the healthcare system, managing feelings and the impact of the illness on social life, work and relationships. We used the Corbin & Strauss model again in the development of the HDSO programme, due to its relevance to the aim of the programme. We linked Corbin & Strauss’ three self-management tasks to the intended outcomes of the HDSO programme (improved knowledge, self-efficacy and emotional wellbeing), and developed a causal model for the intervention (see Appendix 1). In order to achieve the intended behavioural outcomes, behaviour change techniques (BCTs) based on behaviour change theory were used to target key behaviours. BCTs including goal-setting; action planning; and reviewing goals can be seen in the causal model and were key features of the programme. These BCTs have their basis in social cognitive theory,^21^ which suggests that behaviour is influenced by personal factors like self-efficacy (confidence in one’s own capabilities). Goal-setting, action planning and reviewing goals improve self-efficacy by allowing people to monitor and review their progress. When people can see their progress it provides a sense of accomplishment which sustains motivation and skills development.^22–24^

A theory of implementation known as the Normalization Process Theory (NPT) was also considered from the outset of the development of HeLP-Diabetes and HDSO to ensure that they were optimally “implementable” within the NHS.^25^ NPT provides an empirically grounded model of the factors that promote or inhibit the routine incorporation of interventions in everyday practice.^26^ These factors include making sure that the programme was easy to differentiate from other programmes and had clear benefits; fit with professional priorities (such as adhering to NICE recommendations); fit into existing working practices easily; and made consultations between patients and health professionals more productive.^25^

The use of theory and evidence is recommended by the Medical Research Council (MRC) for health researchers developing and evaluating complex interventions. An interdisciplinary approach was taken in the development of the HDSO programme, and methods common to computer science and Human-Computer Interaction (HCI) were used in addition to methods from health. Findings from focus groups undertaken to establish user requirements for the HeLP-Diabetes website (an integral part of the design cycle in HCI research) were combined with the theoretical model described above to develop the first iteration of HeLP-Diabetes: Starting Out. We then carried out usability testing and “in the wild testing” to refine the intervention. These steps are described in more detail elsewhere, along with a detailed description of the initial design, usability testing, “in the wild testing” and revisions.^27^

### What

#### Materials used to deliver intervention

The programme was completed online, and was developed for use either on a tablet or a desktop computer. People therefore needed access to the internet and a tablet or desktop computer to use the programme. People who did not have home access to the internet or a computer, were given information about public internet access at local libraries.^28^

Information about the HDSO programme was given to people with T2DM registered at GP practices in the Clinical Commissioning Groups (CCGs) who commissioned the programme. Information included an outline of the programme and contact details for the HDSO team. People were asked to contact the HDSO team if there were interested in using the programme. The HDSO team registered people and gave them the webpage details (Uniform Resource Locator, or URL) for the programme, and a username and password for accessing it online. The HDSO team and registration process are described further below.

#### Information provided to participants (programme content)

The content of the HDSO programme was a small subset of the HeLP-Diabetes website, selected for its relevance to newly diagnosed people. In selecting content we prioritised actionable information with support for behaviour change and emotional issues, as the underlying principles of HDSO focused on empowering and enabling patients, through increasing perceived autonomy and competence (self-efficacy). Self-regulation theory states that self-efficacy has a strong impact on thought, affect, motivation, and action.^29^ The programme therefore aimed to encourage behaviour change by increasing self-efficacy (see causal model, Appendix 1).

The criteria for selecting content were based on user needs from qualitative data and the experience of the Diabetes Specialist Nurses (DSNs) in the HDSO team who were trained in facilitating face-to-face education with newly diagnosed patients with T2DM. It was decided that all aspects of diabetes management should be included, to give people a good overview of the disease process, its complications and impact on emotions, relationships and work, and an understanding of the types of treatment that are recommended (both lifestyle changes and medical treatment).^25^ People who registered for the HDSO programme, also had access to both the HDSO programme and the HeLP-Diabetes website via a common homepage.

Information was presented using text, images and videos, with an emphasis on the positive (i.e. what people can do to help themselves and improve their health). Text was written for people with a reading age of 12 to correspond to with 80% of the UK population.^30^ A detailed description of the number and timing of the sessions of the programme is given below (‘When and how much’).

#### Procedures

Patients were able to register for the intervention initially by contacting the HDSO team (by email, telephone or post) who then called them by telephone to register their details. The HDSO team then emailed people the webpage URL, a username and password. Following modifications to the programme (detailed below), people were able to register themselves online by visiting the self-registration page of the website.

People could contact the HDSO team for support via telephone. The HDSO team provided support for problems with registration or using the website. Individualised email feedback and reminders were sent at various stages of the programme (see ‘Tailoring’).

### Who provided

The HDSO team consisted of patients, administrators, General Practitioners, Diabetes Specialist nurses (DSNs) and health and HCI researchers. The patients we worked with had experience of living with T2DM, and the GPs had experience of managing patients with T2DM and referring to structured education. The DSNs were trained in facilitating structured education for patients with T2DM. The health and HCI researchers had experience developing and evaluating other online interventions.

The patients, GPs, DSNs and researchers worked together on the initial development of the programme, and the subsequent testing and refinement. The GPs and DSNs wrote the individualised feedback and reminders. The administrators and DSNs visited practices and met with practice managers, GPs and Nurses to publicise the programme and encourage them to inform patients about the programme. It was the role of the administrators to register people and send username and password details. The administrators, GPs and DSNs provided telephone and email support to people who contacted the team with questions. Telephone support was limited to helping people with technical problems such as lost passwords. Coaching and individual clinical advice was not included.

### How

The programme was delivered online for individuals. Telephone support from an administrator was available for technical problems, such as lost passwords or problems logging on, and individualised feedback and reminders were provided by email. People could interact with other users via an online forum (part of the HeLP-Diabetes website which HDSO users had access to), where they could post messages about their progresses, tips or queries.

### Where

The programme was available to adults with T2DM registered with a practice in one of the Clinical Commissioning Groups (CCGs) that commissioned the programme.

The programme was delivered online, and could therefore be used wherever people had computer and internet access. The HDSO team were based at the Royal Free Hospital Campus of University College London. They provided support to users via telephone and email.

### When and how much

The programme had four “sessions” which were divided into four or five parts or modules. The sessions are described in more detail below, starting with the spiral curriculum which formed the core structure of the programme.

#### Spiral curriculum

The programme followed a spiral curriculum. This means that people built on the knowledge they acquired in each session as they proceeded. The spiral model is an evidence-based model for adult education which is based on the Harden and Stamper spiral curriculum model.^31^ This model was chosen because it allows for an ‘iterative revisiting of topics, subjects or themes throughout the course’.^31^ The idea is that topics are not just repeated, but that knowledge and understanding should be deepened each time. The learner’s competence should increase with each visit until the overall aim is achieved.

**Figure 1.**
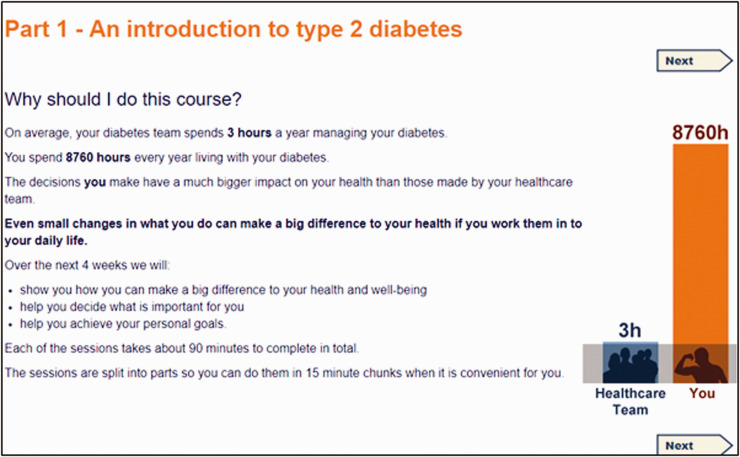
Screenshot of Week 1 Part 1 taken from HeLP-Diabetes: Starting Out.

**Figure 2.**
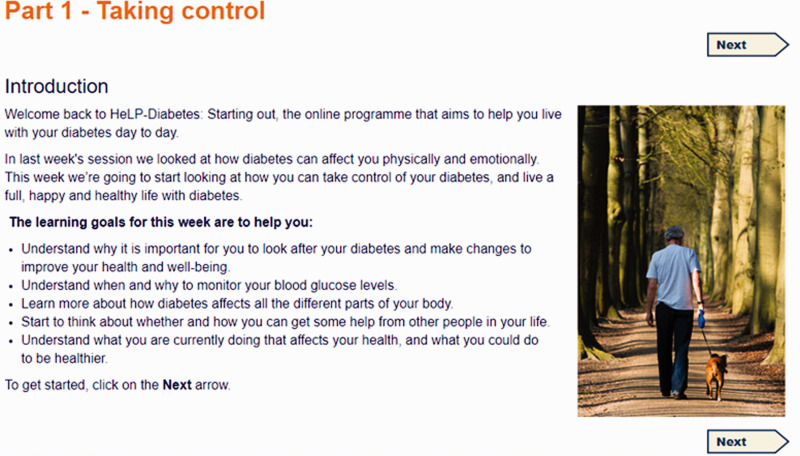
Screenshot of Week 2 Part 1 taken from HelP-Diabetes: Starting Out.

**Figure 3.**
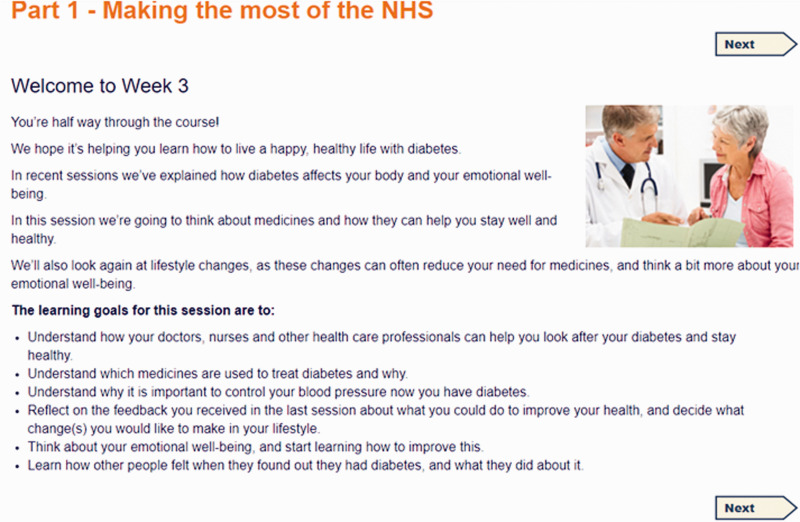
Screenshot of Week 3 Part 1 taken from HeLP-Diabetes: Starting Out.

**Figure 4.**
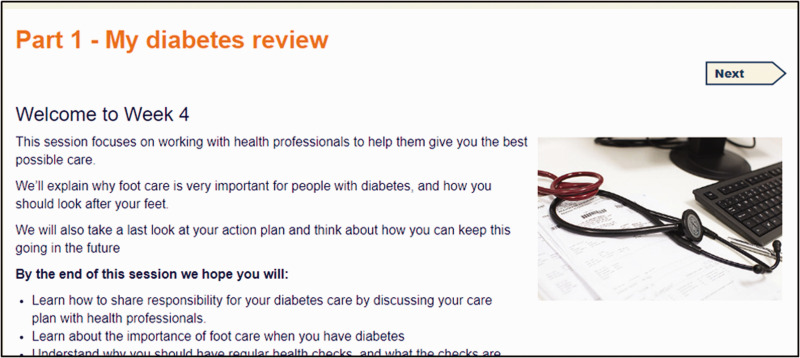
Screenshot of Week 4 Part 1 taken from HeLP-Diabetes: Starting Out.

**Figure 5.**
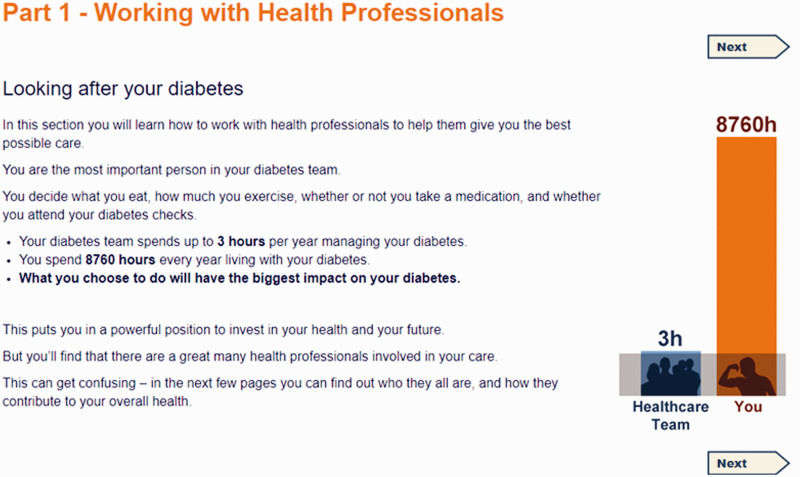
Screenshot of Week 5 Part 1 taken from HeLP-Diabetes: Starting Out.

The curriculum was structured into four sessions, with a fifth bonus session. Each session consisted of four or five parts and took about ninety minutes to complete. The titles of each session and part in listed in Table 3, and illustrated with screenshots (see Figure 1–5). Each session addressed the three domains described by Corbin and Strauss. Sessions could be completed at one sitting, or progress could be saved and users could dip in and out. Users were encouraged to complete one session a week.

#### Session descriptions

##### Week 1 – getting started

Week 1 contained information about what diabetes is (see Figure 1), eating well for diabetes and being active, and self-assessment questionnaires (described in more detail below).

##### Week 2 – self-management

Week 2 Part 1 (‘Taking Control’, see Figure 2) contained information on monitoring blood glucose levels and healthy behaviours, and quizzes on physical activity, medication use, alcohol intake and diet. ‘Protecting my body and mind’ included information about preventing problems with emotions, eyes, feet, infections, kidneys, nerves, sexual function, and abnormal blood sugar levels. ‘Handling feelings’ contained information about how diabetes can affect relationships at work and at home, and videos of people with diabetes talking about how they approached these issues. ‘Making changes’ was an exercise for reflecting on the self-assessment questionnaires in Week 1, and setting specific, measurable, achievable, realistic and time-bound (SMART) goals for diet, medicine, activity, drinking and other health behaviour changes.

##### Week 3 – improving my health and wellbeing

Week 3 Part 1 (‘Making the most of the NHS’, see Figure 3) provided information about the essential checks that all people with diabetes should receive, videos of people talking about their interaction with the NHS, and a link to the health record in HeLP-Diabetes where users can record appointments). ‘Medication’ included information about the purpose of medication, videos about the challenges and benefits of medications, advice for concerns about medications, a “My medicines” list, and information about commonly used medications in diabetes. ‘Reducing the risks of heart attack and stroke’ was an explanation of the importance of blood pressure, how it is measured, and heart disease and its treatment and prevention. ‘Update my goals and plans’ was a look back to the SMART goals. ‘Understanding my moods’ was a series of videos of people with diabetes explaining how they felt when they found out they had diabetes, and what they did to make themselves feel better.

##### Week 4 – taking control of my diabetes

Week 4 Part 1 was ‘’My diabetes review’ (see Figure 4), a series of videos about people’s experiences of diabetes care, and explanation of the diabetes care plan. ‘Looking after my feet’ explained the different types of foot problems that people with diabetes can experience, how to prevent foot problems, foot checks and tests, and foot complications from diabetes and their management. ‘Review my goals and plans’ was another opportunity to review and update SMART goals. ‘Self-assessment’ was the end of programme self-assessment questionnaires. ‘Moving on: the beginning of the end’ was advice about staying motivated and links to further information.

##### Week 5 – bonus content

Week 5 contained supplementary content (see Figure 5). This information included working with health professionals, managing diabetes when ill, diabetes and social life, work, driving, reviewing goals and plans, and more on managing moods.

### Tailoring

#### Self-assessment

Personalized feedback was sent to users who completed the self-assessment questionnaires. Self-assessment questionnaires were included in Weeks 1 and 4 of the HDSO programme. Self-assessment has been identified as key to managing one’s motivation towards learning by education theorists.^32–34^ Self-assessment allows acknowledgement of pre-existing understanding, and along with feedback, can help people focus on their learning needs.^35^ The questionnaires were positioned to help people recognise their learning needs at the start of the programme, and then reflect on their overall learning and progress at the end of the programme. Anonymised questionnaire scores were also used to provide evidence of impact of the intervention for research.^19^

At Week 1 the questionnaires were used as formative assessments. Formative practice allows learners to understand their strengths and weaknesses, in order to make progress.^36^ For an assessment to be formative, it requires feedback which indicates whether there is a gap in learning, how learning can be improved (see Appendix 1).^37^ The aim of the Week 1 questionnaires was to help people recognise their learning needs at the start of the programme, and to make clear the purposes and goals of the programme. Feedback at this stage helped learners to understand how they could improve by signposting them to parts of the course that would help address the gaps in their learning.

At week 4, the questionnaires were used as summative assessments. Summative assessment is a recording of the overall achievement of the student, and relates to progression in their learning.^36^ Formative assessment can be seen as helping learning to move forward, whereas summative assessment can be seen as summarizing learning that has taken place.^38^ The aim of the Week 4 questionnaires was to help people to reflect on the learning they had accomplished by the end of the programme. Feedback was provided to summarise the learning and achievement that had taken place.

The questionnaires that were used were the: (1) Audit of Diabetes Knowledge (AdKnowl); (2) Diabetes Management Self-efficacy Scale (DMSES); and (3) Problem Areas in Diabetes (PAID). These questionnaires were chosen based on the outcomes proposed from the causal model of the intervention (see Appendix 1, and^27^) We linked the theoretical framework (the self-management tasks proposed by Corbin and Strauss) with the components of the intervention, to predict a range of effects. The proximal effects that were proposed were improved knowledge, increased motivation, increased self-efficacy, and decreased emotional distress. More distal effects predicted were weight loss and increased physical activity, with final outcomes being improved HbA1c and health-related quality of life. We chose to measure the proximal outcomes because it was more feasible to measure knowledge, self-efficacy and diabetes-related emotional distress (they could all be measures using online self-assessment questionnaires built into the HDSO programme); because there were reliable and valid questionnaires available to measure these outcomes; and because improving these outcomes made it more likely (as predicted by behaviour change theory) that people would be able to change distal outcomes like weight loss and physical activity.

##### Knowledge

The Audit of Diabetes Knowledge (Adknowl) was chosen to assess knowledge. It was chosen because one of its purposes is to evaluate the success of educational interventions. There are 23 item-sets (114 items), all of which reflect the content of the HDSO programme. These are: diabetes treatment, sick days, hypoglycaemia, complication risk, physical activity, smoking, alcohol, foot care and diet. Higher scores indicate greater knowledge and the topic sub-sets allow specific knowledge deficits to be targeted.^39^ This meant that knowledge deficits could be highlighted to users, as well as advice about which parts of the course could help address these deficits.

##### Self-efficacy

The Diabetes Management Self-Efficacy Scale (DMSES) was used to measure self-efficacy. It has 20 items and measures individuals’ expectations for being able to engage in diabetes self-management activities which reflect activities targeted by the HDSO programme, like daily exercise, and keeping to a healthy eating plan when away from home. The questionnaire has been used in previous research, and has been found to have good internal reliability, criterion and construct validity, and acceptable test-retest reliability.^40^

##### Emotional distress

Problem Areas in Diabetes (PAID) was used to measure diabetes-related emotional distress. It has 20 items focusing on areas that cause difficulty for people living with diabetes, including social situations, food, friends and family and social support. Psychometric tests have shown that PAID has consistently high internal reliability, sound test-retest reliability, and was a statistically significant predictor of glycaemic control in a one-year study of a managed care population.^41,42^

#### Personalized feedback

Feedback has been described above as being a necessary part of self-assessment as it can provide information on gaps in learning and instruction on how learning can be improved. Users were emailed their actual scores for knowledge, self-efficacy and distress about diabetes self-management, with total possible scores, as personalised feedback. The total possible scores provided people with reference levels for knowledge and skills. In order to help people move from their actual levels to reference levels of knowledge and skills, at Week 1 we signposted users to parts of the programme that would help them improve. This is illustrated in Table 4. This is proposed in the literature by Ramaprasad and Sadler as a way of giving effective feedback.^34,43^

At Week 4, feedback was provided to record achievement and progress in learning. When scores improved, users were congratulated. When these parameters stayed the same, users were reassured that their self-efficacy, distress and knowledge would improve as they learned more about diabetes self-management. When the parameters did not improve, we explained why this can occur. For example, self-efficacy can decrease and distress can increase with greater awareness of complications. Knowledge can apparently decrease because the increase in the amount of information the user is exposed to may cause confusion. This fits with theory on cognitive overloading, which suggests that performance deteriorates with excessively high cognitive load.^44^ Users who had a decrease in knowledge score were reassured that making sense of all the information would take time, and any topics of confusion could be clarified by referring to other sources, including the main HeLP-Diabetes programme, peer support groups, face-to-face group education programmes and scientific research papers. These were all described and links were given to websites with further information in Week 4 Part 5 (“Moving on: the end of the beginning”).

#### Personalized emails

Personalized emails were sent to users to promote engagement and maintain motivation. Systematic reviews of strategies to improve adherence to digital health interventions provide some evidence of effect for email reminders.^45,46^ Alkhaldi et al. found that studies of the effectiveness of prompts to increase of digital interventions reported borderline small-to-moderate positive effects of technological strategies, including emails, to improve use of interventions.^46^ As we were mindful of the need for the intervention to be scalable if it were to be rolled out across the NHS, emails were chosen as a cost and time-effective strategy, compared to telephone or face-to-face contact.

Emails were sent automatically if: (i) a user completed a session or; (ii) a user did not log in to the programme for a week or more. Emails were personalised by addressing users by their names. The emails sent on completion of a session included a congratulations message to acknowledge and monitor progress, and an outline of the topic of the next part of the programme. People who did not log in to the programme for one week received an email reminder that the programme was open to them, and a brief outline of the content of the next part of the programme. This was repeated if the user did not login for two weeks, and again if they did not login for three weeks. Emails did not include coaching or individual clinical advice.

#### Goal-setting, action planning and reviewing goals

The overall programme included support for behaviour change in the areas of diet, physical activity, taking medication, smoking cessation and moderating alcohol consumption. Goal-setting, action planning and reviewing goals were behaviour change techniques (BCTs) used to tailor the intervention and promote behaviour change. BCTs are based on theories of behaviour change, which predict how behaviour change occurs.^47^ Studies of digital health behaviour change interventions have found that interventions that use more BCTs have larger effects compared to interventions that use fewer techniques.^48^ In light of this evidence the BCTs identified as effective for changing the behaviours relevant to type 2 diabetes (goal-setting, action planning and reviewing goals) were adopted in the HDSO programme. . This is described in more detail below.

In Week 2 of the programme, users were asked to set specific, measurable, achievable, realistic, time-bound (SMART) goals.^49^ The rationale for asking users to choose specific achievable goals is based on theories of self-efficacy,^50^ and goal-setting is used in other diabetes self-management programmes.^51^ Goal-setting increases motivation, as it allows us to compare our performance against personal standards we have set for ourselves.^22^ Easier, more proximal and specific goals are easier to monitor and more likely to be achieved.^23,52^

**Figure 6.**
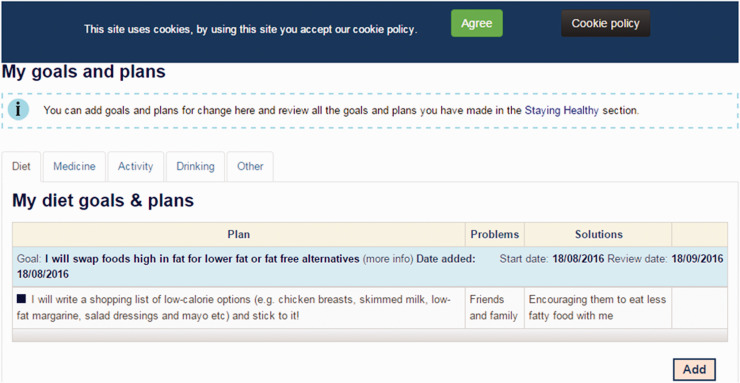
Screenshot of goal-setting exercise.

Users could enter and save their goal onto the website (see Figure 6), along with a plan of exactly what they were going to do (e.g. stick to a diet goal by taking a shopping list of low calorie food to the supermarket), potential barriers, and ideas for navigating these barriers. People could also select a choice of email or text reminder and a review date, to help them monitor and achieve their goals.

Later in the course there were opportunities to review goals that had been set, and rate progress from 0 to 5. People were asked questions about how they felt about their progress, and given encouragement and feedback such as making a goal more achievable, or setting new reminders.

### Modifications

This paper has described the final intervention that resulted after an iterative process of evaluation and modification. This section describes the modifications which were undertaken in order to produce the final intervention.

The evaluation process involved usability testing and “in the wild” testing in the NHS.^27^ The quantitative findings from these evaluations suggested that there were problems with uptake and completion of the programme. The qualitative findings suggested that there were both patient and programme factors influencing uptake and completion. We used the qualitative findings to inform modifications that we made to the programme. These are summarised below:

1. Reducing the number of session from eight to four. The programme was shortened due to people’s concerns that it would take too long, or that they did not have enough time to complete it. All aspects of management of type 2 diabetes were retained. Topics relating to special situations such as ‘Managing my diabetes when I’m ill’, and ‘Working with diabetes’, were moved to the fifth bonus session.
2. Reducing the number of questionnaires from three to two. The AdKnowl (knowledge) questionnaire^39^ was removed because it was significantly longer than the other two questionnaires anduser feedback suggested patients found it burdensome and off-putting.
3. Online self-registration was introduced to reduce the time it took patients to access the programme. People who contacted the HDSO team were emailed the URL of the self-registration page. Telephone support was still available for those who had difficulty.

We also made the programme available to everyone with T2DM, not just those who were newly diagnosed. We based this decision not just on uptake and completion data, but also on data from the National Diabetes Audit (NDA) which shows that not all patients are offered structured education at the time of diagnosis, and of those who are offered it, in 2016–7 only 7.1% attended.^53^ There are therefore many patients with T2DM who are not newly diagnosed but have not received structured self-management education, and are in need of it. It was therefore decided to offer it everyone with T2DM, but to compare the uptake and completion rates between patients who were newly diagnosed and those who were not.

#### How well

Intervention adherence and fidelity were assessed by the HDSO team in a mixed methods study of feasibility, acceptability and impact. The processes and outcomes of this study are outside the scope of this paper, and are described in detail elsewhere.^19^

## Discussion

This paper uses the TIDieR checklist to describe the HeLP-Diabetes: Starting Out programme. Items in the checklist include the theoretical underpinning, materials, procedures, providers, mode of delivery, structure, dose, personalisation and modifications of the intervention. Descriptions of interventions are important because they allow researchers to replicate and build on existing knowledge. In the field of behaviour change research, reviews have found that reporting of interventions in published evaluations is insufficient to reliably identify content and effective ingredients.^54,55^ Descriptions of content are usually brief and vague, using broad categorisations such as ‘behavioural counselling’ or ‘motivational strategies’. In some cases, studies describe mode of intervention delivery such as ‘face to face’ or ‘nurse delivered’, but don’t mention content. In the field of T2DM, reviews of online T2DM self-management interventions have highlighted problems with lack of high quality descriptions of interventions. Greenwood et al conducted a review of reviews of technology-enabled diabetes self-management education and support, and found that education was described as either generalised, customised or a combination of the two, but poorly defined in more detail.^15^ The authors also found that feedback was described as live or automated, but the type of feedback that was given was not described.^15^ A 2013 Cochrane review of computer-based diabetes self-management interventions for adults with T2DM also found that interventions were not described in sufficient detail to replicate them.^8^ The authors found that this limited the possibility of specifying component behaviour change techniques, or identifying the likely mechanisms of action.

The aim of this paper was to address some of these gaps in the literature, particularly on online interventions for T2DM self-management, by providing a detailed description of the intervention. Our description is novel in that it includes screenshots and summaries of the content of each session of the programme, and the details and rationale for BCTs and personalised components. This description serves to highlight the some of the strengths of the programme, which include the scope of information and the way information is presented (in text with an appropriate reading age, video and images); the use of BCTs with evidence for effectiveness in behaviour change interventions (goal-setting and action-planning); and tailoring of feedback and email reminders to help users work out their level of knowledge and skills and how to improve. Modifications were made to the intervention after data were collected from usability testing and “in the wild testing”, to help improve uptake and completion. Further modifications could be made to the intervention to improve uptake and completion, such as mobile access via a smartphone application, and more regular email reminders to programme users with an increased amount of personalisation.

The TIDieR checklist was chosen as a template for the description of the intervention, because of the clear structure it uses and the inclusion of items relevant to an online structured education programme (including procedures, mode of delivery and dose). The TIDieR checklist allowed us to provide a clear structure for this paper, with items from the checklist used as section headings (‘Brief name’, ‘Why’, ‘What’, etc). The developers of the TIDieR checklist have acknowledged that describing complex interventions is challenging, and a checklist cannot capture the full complexity of some interventions.^16^ This was the case for the description of HDSO, and subcategories needed to be added to some of the items from the checklist to capture the intervention more accurately. HDSO was a structured education programme, and so additional subcategories were particular necessary when it came to describing the programme content and structure. Hence we added subcategories to the ‘What’ and ‘When and how much items’ to include ‘Information provided to participants (programme content)’, ‘Spiral curriculum’ and ‘session descriptions’. The checklist provided an appropriate overall template for the description, but intervention developers may find that it needs to be adapted to make it more specific for particular interventions.

## Conclusions

With this paper we have attempted to provide a description of the HeLP-Diabetes: Starting Out online structured education programme of sufficient detail, that researchers will be able to use to replicate and build on their interventions. It is hoped that this will help improve the quality and uptake of similar interventions in the future.

## Supplemental Material

sj-pdf-1-dhj-10.1177_2055207620975647 - Supplemental material for Use of the TIDieR checklist to describe an online structured education programme for type 2 diabetes Click here for additional data file. Supplemental material, sj-pdf-1-dhj-10.1177_2055207620975647 for Use of the TIDieR checklist to describe an online structured education programme for type 2 diabetes by Shoba Poduval, Jamie Ross, Kingshuk Pal, Nicola Newhouse, Fiona Hamilton and Elizabeth Murray in Digital Health

## References

[1] Diabetes UK Patient Education Working Group. *Structured patient education in diabetes.* London: Department of Health and Diabetes UK, 2005.

[2] Tomky D Tomky D Cypress M, et al Aade position statement. The Diabetes Educator 2008; 34: 445–449.18535317

[3] National Institute for Health and Clinical Excellence. *Type 2 Diabetes in adults: management* NICE guideline NG28 2015, https://www.nice.org.uk/guidance/ng28 (accessed 10 November 2020).

[4] NHS Digital. National Diabetes Audit, 2016-17 Report 1: Care Processes and Treatment Targets 2018, https://files.digital.nhs.uk/pdf/s/k/national_diabetes_audit_2016-17_report_1__care_processes_and_treatment_targets.pdf (accessed 10 November 2020).

[5] Horigan G Davies M Findlay-White F, et al Reasons why patients referred to diabetes education programmes choose not to attend: a systematic review. Diabet Med 2017; 34: 14–26. 26996982 10.1111/dme.13120

[6] Diabetes UK. *Diabetes education: the big missed opportunity in diabetes care* London: Author, 2015.

[7] All-Party Parliamentary Group for Diabetes. *Taking control: supporting people to self manage their diabetes*. London, All-Party Parliamentary Group for Diabetes, 2015.

[8] Pal K Eastwood SV Michie S, et al Computer-Based Interventions to Improve Self-management in Adults With Type 2 Diabetes: A Systematic Review and Meta-analysis. *Diabetes Care* 2013; 37: 1759. 10.2337/dc13-1386 24855158

[9] Pereira K Phillips B Johnson C, et al Internet delivered diabetes self-management education: a review. Diabetes Technol Ther 2015; 17: 55–63. 25238257 10.1089/dia.2014.0155

[10] Ramadas A Quek KF Chan CK, et al Web-based interAventions for the management of type 2 diabetes mellitus: a systematic review of recent evidence. Int J Med Inform 2011; 80: 389–405. 21481632 10.1016/j.ijmedinf.2011.02.002

[11] van Vugt M de Wit M Cleijne WH, et al Use of behavioral change techniques in web-based self-management programs for type 2 diabetes patients: systematic review. J Med Internet Res 2013; 15: e279. 24334230 10.2196/jmir.2800 PMC3869055

[12] Murray E Sweeting M Dack C, et al Web-based self-management support for people with type 2 diabetes (HeLP-Diabetes): randomised controlled trial in English primary care. BMJ Open 2017; 7: e016009. 10.1136/bmjopen-2017-016009 PMC5623569 28954789

[13] Quality Institute for Self-Management Education. DSME Standard, www.qismet.org.uk (2010, accessed 14 February 2019).

[14] Michie S Fixsen D Grimshaw JM, et al Specifying and reporting complex behaviour change interventions: the need for a scientific method. Implement Sci 2009; 4: 40. 19607700 10.1186/1748-5908-4-40 PMC2717906

[15] Greenwood DA Gee PM Fatkin KJ, et al A systematic review of reviews evaluating technology-enabled diabetes self-management education and support. J Diabetes Sci Technol 2017; 11: 1015–1027. 28560898 10.1177/1932296817713506 PMC5951000

[16] Hoffmann TC Glasziou PP Boutron I, et al Better reporting of interventions: template for intervention description and replication (TIDieR) checklist and guide. BMJ 2014; 348: g1687. 24609605 10.1136/bmj.g1687

[17] Dack C Ross J Stevenson F, et al A digital self-management intervention for adults with type 2 diabetes: combining theory, data and participatory design to develop HeLP-Diabetes. Internet Interv 2019; 17: 100241. 31372349 10.1016/j.invent.2019.100241 PMC6660456

[18] Hoffmann T English T Glasziou P. Reporting of interventions in randomised trials: an audit of journal instructions to authors. Trials 2014; 15: 20. 24422788 10.1186/1745-6215-15-20 PMC3896798

[19] Poduval S, Marston L, Hamilton F, et al. Feasibility, Acceptability, and Impact of a Web-Based Structured Education Program for Type 2 Diabetes: Real-World Study. *JMIR Diabetes* 2020; 5: e15744. 10.2196/15744 PMC6971513 31904580

[20] Corbin JM Strauss A. Unending work and care: Managing chronic illness at home. San Francisco: Jossey-Bass, 1988.

[21] Bandura A. *Social foundations of thought and action* Englewood Cliffs, NJ: New Jersey, Prentice Hall, 1986.

[22] Bandura A Schunk DH. Cultivating competence, self-efficacy, and intrinsic interest through proximal self-motivation. J Personal Soc Psychol 1981; 41: 586–598.

[23] Schunk DH. Goal setting and self-efficacy during self-regulated learning. Educ Psychologist 1990; 25: 71–86.

[24] Elliott ES Dweck CS. Goals: an approach to motivation and achievement. J Pers Soc Psychol 1988; 54: 5–12. 3346808 10.1037//0022-3514.54.1.5

[25] Murray E, Ross J, Pal K, et al. A web-based self-management programme for people with type 2 diabetes: the HeLP-Diabetes research programme including RCT. *Programme Grants for Applied Research* 2018. 30199193

[26] May CR Finch T Ballini L, et al Evaluating complex interventions and health technologies using normalization process theory: development of a simplified approach and web-enabled toolkit. BMC Health Serv Res 2011; 11: 245. 21961827 10.1186/1472-6963-11-245 PMC3205031

[27] Poduval S Ross J Pal K, et al interdisciplinary research approach to the development and formative evaluation of an online structured education programme for type 2 diabetes. *Submitted.*

[28] GOV.UK. Book internet access in your library 2019, https://www.gov.uk/book-internet-access-at-library (accessed August 2019).

[29] Bandura A. Social cognitive theory of self-regulation. Org Behav Human Decision Process 1991; 50: 248–287.

[30] National Literacy Trust. Adult literacy 2017, https://literacytrust.org.uk/parents-and-families/adult-literacy/ (accessed 3 January 2018). (Archived by WebCite® at http://www.webcitation.org/6yzfl2B0J.)

[31] Harden RM. What is a spiral curriculum? Med Teach 1999; 21: 141–143. 21275727 10.1080/01421599979752

[32] Black P McCormick R James M, et al Learning how to learn and assessment for learning: a theoretical inquiry. Res Papers Educ 2006; 21: 119–132.

[33] Wiliam D Thompson M. Integrating assessment with instruction: what will it take to make it work? In CA Dwyer (ed.) *The future of assessment: shaping teaching and learning*. Mahwah, NJ: Erlbaum (ND), 2007, pp. 53–82.

[34] Ramaprasad A. On the definition of feedback. Behav Sci 1983; 28: 4–13.

[35] Dweck CS. Self-theories: Their role in motivation, personality, and development. Florence, KY: Psychology Press, 2000.

[36] Harlen W James M. Assessment and learning: differences and relationships between formative and summative assessment. Assess Educ Principles Policy Pract 1997; 4: 365–379.

[37] Taras M. Assessment – summative and formative – some theoretical reflections. Brit J Educ Stud 2005; 53: 466–478.

[38] Harlen W. Teachers' summative practices and assessment for learning – tensions and synergies. Curriculum J 2005; 16: 207–223.

[39] Speight J Bradley C. The ADKnowl: identifying knowledge deficits in diabetes care. Diabet Med 2001; 18: 626–633. 11553199 10.1046/j.1464-5491.2001.00537.x

[40] Sturt J Hearnshaw H Wakelin M. Validity and reliability of the DMSES UK: a measure of self-efficacy for type 2 diabetes self-management. Primary Health Care Res Dev 2010; 11: 374–381.

[41] Snoek F Welch G. Problem Areas in Diabetes (PAID) questionnaire. Denmark: Novo Nordisk, 2006.

[42] Welch G, Weinger K, Anderson B and Polonsky WH. Responsiveness of the Problem Areas In Diabetes (PAID) questionnaire. *Diabet Med* 2003; 20: 69–72. 10.1046/j.1464-5491.2003.00832.x 12519323

[43] Black P Wiliam D. Assessment and classroom learning. Assess Educ Principles Policy Pract 1998; 5: 7–74.

[44] Teigen KH. Yerkes-Dodson: a law for all seasons. Theor Psychol 1994; 4: 525–547.

[45] Brouwer W Kroeze W Crutzen R, et al Which intervention characteristics are related to more exposure to internet-delivered healthy lifestyle promotion interventions? A systematic review. J Med Internet Res 2011; 13: e2. 21212045 10.2196/jmir.1639 PMC3221341

[46] Alkhaldi G Hamilton LF Lau R, et al The effectiveness of prompts to promote engagement with digital interventions: a systematic review. J Med Internet Res 2016; 18: e6. 26747176 10.2196/jmir.4790 PMC4723726

[47] Webb TL Joseph J Yardley L, et al Using the internet to promote health behavior change: a systematic review and meta-analysis of the impact of theoretical basis, use of behavior change techniques, and mode of delivery on efficacy. J Med Internet Res 2010; 12: e4. 20164043 10.2196/jmir.1376 PMC2836773

[48] Webb LT Joseph J Yardley L, et al Using the internet to promote health behavior change: a systematic review and meta-analysis of the impact of theoretical basis, use of behavior change techniques, and mode of delivery on efficacy. J Med Internet Res 2010; 12: e4. 20164043 10.2196/jmir.1376 PMC2836773

[49] Doran G. There's a SMART way to write management's goals and objectives. Manag Rev 1981; 70: 35–36.

[50] Bandura A. Self-efficacy: toward a unifying theory of behavioral change. Psychol Rev 1977; 84: 191–215. 847061 10.1037//0033-295x.84.2.191

[51] Khunti K Gray LJ Skinner T, et al Effectiveness of a diabetes education and self management programme (DESMOND) for people with newly diagnosed type 2 diabetes mellitus: three year follow-up of a cluster randomised controlled trial in primary care. BMJ 2012; 344: e2333–e2333. 22539172 10.1136/bmj.e2333 PMC3339877

[52] Schunk DH. Self-efficacy and academic motivation. Educ Psychologist 1991; 26: 207–231.

[53] NHS Digital. National Diabetes Audit, 2015–2016. Report 1: care processes and treatment targets, https://digital.nhs.uk/catalogue/PUB23241. (2017, accessed 31 Jan 2018).

[54] Glasziou P Meats E Heneghan C, et al What is missing from descriptions of treatment in trials and reviews? 2008 336:1472–1474. 10.1136/bmj.39590.732037.47 PMC2440840 18583680

[55] Michie S Abraham C Whittington C, et al Effective techniques in healthy eating and physical activity interventions: a meta-regression. Health Psychol 2009; 28: 690–701. 19916637 10.1037/a0016136

