# Supplementary material for: Use of the TIDieR checklist to describe an online structured education programme for type 2 diabetes
Source: Digit Health. 2020 Nov 30;6:2055207620975647. doi: 10.1177/2055207620975647 (PMC7708660; doi:10.1177/2055207620975647)
Supplement: sj-pdf-1-dhj-10.1177_2055207620975647 - Supplemental material for Use of the TIDieR checklist to describe an online structured education programme for type 2 diabetes [file sj-pdf-1-dhj-10.1177_2055207620975647.pdf]

Causal Model of HDSO

<table>
<tr><th colspan="12">Self-management tasks faced by people with chronic conditions</th></tr>
<tr><td colspan="4">Role management</td><td colspan="4">Medical management</td><td colspan="4">Emotional management</td></tr>
<tr><th colspan="12">Intervention components</th></tr>
<tr><td colspan="3">Information provision
An introduction to diabetes
Protecting my mind and body Reducing the risks of heart attacks & strokes
Having a social life
Driving</td><td colspan="3">Goal setting and action planning
Self-assessment and feedback
My diet and physical activity goal; Update my goals and plans
self-assessment and feedback
Review my goals and plans.</td><td colspan="3">Behaviour Change support
Eating well for diabetes
Becoming more active
Medication
Looking after my feet.</td><td colspan="3">Emotional support
Protecting my mind and body
Handling feelings
Understanding my moods</td></tr>
<tr><th colspan="12">Targets</th></tr>
<tr><td colspan="2">Motivation</td><td colspan="2">Knowledge</td><td colspan="2">Self-efficacy</td><td colspan="2">Intentions</td><td colspan="2">Behaviour</td><td colspan="2">Emotions</td></tr>
<tr><th colspan="12">Proximal Outcomes</th></tr>
<tr><td colspan="6">Improved knowledge</td><td colspan="6">Increased motivation & self-efficacy & decreased emotional distress</td></tr>
<tr><th colspan="12">Distal Outcomes</th></tr>
<tr><td colspan="12">Weight loss, increased physical activity, improved HbA1c and Health-related Quality of Life.</td></tr>
</table>
